# Biomimetic Computing for Efficient Spoken Language Identification

**DOI:** 10.3390/biomimetics10050316

**Published:** 2025-05-14

**Authors:** Gaurav Kumar, Saurabh Bhardwaj

**Affiliations:** Department of Electrical and Instrumentation Engineering, Thapar Institute of Engineering and Technology, Patiala 147001, India; saurabh.bhardwaj@thapar.edu

**Keywords:** spoken language identification, dung beetle optimization, long short-term memory, Bayesian optimization, deep learning, multi-class spoken language identification

## Abstract

Spoken Language Identification (SLID)-based applications have become increasingly important in everyday life, driven by advancements in artificial intelligence and machine learning. Multilingual countries utilize the SLID method to facilitate speech detection. This is accomplished by determining the language of the spoken parts using language recognizers. On the other hand, when working with multilingual datasets, the presence of multiple languages that have a shared origin presents a significant challenge for accurately classifying languages using automatic techniques. Further, one more challenge is the significant variance in speech signals caused by factors such as different speakers, content, acoustic settings, language differences, changes in voice modulation based on age and gender, and variations in speech patterns. In this study, we introduce the DBODL-MSLIS approach, which integrates biomimetic optimization techniques inspired by natural intelligence to enhance language classification. The proposed method employs Dung Beetle Optimization (DBO) with Deep Learning, simulating the beetle’s foraging behavior to optimize feature selection and classification performance. The proposed technique integrates speech preprocessing, which encompasses pre-emphasis, windowing, and frame blocking, followed by feature extraction utilizing pitch, energy, Discrete Wavelet Transform (DWT), and Zero crossing rate (ZCR). Further, the selection of features is performed by DBO algorithm, which removes redundant features and helps to improve efficiency and accuracy. Spoken languages are classified using Bayesian optimization (BO) in conjunction with a long short-term memory (LSTM) network. The DBODL-MSLIS technique has been experimentally validated using the IIIT Spoken Language dataset. The results indicate an average accuracy of 95.54% and an F-score of 84.31%. This technique surpasses various other state-of-the-art models, such as SVM, MLP, LDA, DLA-ASLISS, HMHFS-IISLFAS, GA base fusion, and VGG-16. We have evaluated the accuracy of our proposed technique against state-of-the-art biomimetic computing models such as GA, PSO, GWO, DE, and ACO. While ACO achieved up to 89.45% accuracy, our Bayesian Optimization with LSTM outperformed all others, reaching a peak accuracy of 95.55%, demonstrating its effectiveness in enhancing spoken language identification. The suggested technique demonstrates promising potential for practical applications in the field of multi-lingual voice processing.

## 1. Introduction

Spoken language identification is the process of identifying the language spoken from a given speech signal. SLID plays an important role in multi-lingual speech recognition, diarization, and in machine translation systems [1]. Call centers utilize this technology to automatically route incoming calls to operators who are native speakers of the caller’s language [2]. Traditional SLID methods can be grouped on the basis of different features—namely, acoustic, phonotactic, prosodic, and lexical–syntactic [3]. Among these, acoustic features are typically the first layer of speech analysis, extracted directly from the raw speech signal during the parameterization process. Phonotactic features represent a more abstract level of information, capturing the allowable sequences of phonemes specific to a language. These phonotactic rules reflect the structural constraints on how sounds can be combined within that language. Prosodic features—such as intonation, stress, rhythm, and duration—carry useful information, they are not commonly exploited in SLID systems. Languages also have distinct vocabularies, word formation rules, and syntactic structures. These linguistic elements allow for language identification to be conducted at the word or syntax level. Over the past decade, significant advancements have been made in the field of SLID. Deep learning-based frameworks provide researchers with new avenues for exploration and development. Recently, there has been an increasing apprehension about language detection in complex and real-world scenarios [4]. The main step in SLID is feature extraction, which entails finding the relevant speech signal features that are crucial for detecting linguistic content, while simultaneously eliminating redundant information and background noise [5]. For effective SLID, the extracted feature set should exhibit characteristics such as long-term stability, low dimensionality, resilience to noise, and independence from other features. Therefore, the development of robust feature extraction methods remains a significant challenge in the field of automatic SLID [6].

The development of an algorithm for SLID has significantly improved human–system interaction [7]. Extensive research has been conducted in this field, focusing on various techniques for accurate language identification [8]. Researchers initially analyzed speech signals as continuous inputs without incorporating their first- and second-order derivatives. While high-level methods, such as prosody and phonotactics, serve as key sources for language detection, acoustic modeling remains a fundamental component of the process [9].

Feature extraction plays a critical role in pattern recognition, as it involves computation and selection relevant features from speech signals. Among the widely used techniques, Mel-Frequency Cepstral Coefficients (MFCC) have demonstrated exceptional performance in speech and audio processing. The effectiveness of language identification depends on several factors, including the amount of linguistic data, specific application requirements, and the availability of computational facilities [10]. Despite notable advancements in language identification, significant challenges remain, particularly for languages with limited resources.

The present study introduces the Dung Beetle Optimization Deep Learning for Multi-Class Spoken Language Identification System (DBODL-MSLIS) technique, which integrates biomimetic optimization strategies with deep learning to improve the accuracy and efficiency of SLID. This technique draws inspiration from the natural foraging behavior of dung beetles, which navigate their environment to locate and transport resources. In the context of SLID, the proposed method optimizes feature selection and classification processes to improve accuracy and efficiency in distinguishing multiple languages. The process begins with speech preprocessing, which includes steps such as pre-emphasis, windowing, and frame blocking. During this stage, speech signals undergo analysis to extract key features, including pitch, energy, Discrete Wavelet Transform (DWT), and Zero Crossing Rate (ZCR).

SLID has been a widely researched area with various techniques to enhance its accuracy and efficiency. The accuracy of the SLID model is highly sensitive to the duration of speech and amount of information. In order to meet the challenges involved in identification tasks, a highly accurate and computationally efficient framework of i-vector extraction was proposed by Maarten Van Segbroeck et al. in 2015 [11]. This rapid language identification system extracts acoustic features of the spoken languages and deploys an i-vector based framework to discriminate between the target languages of interest. Reference [12] provides an approach for detecting spoken language. The technique presented utilizes a hybrid Convolutional Neural Network (CNN) known as CRNN, which combines a CNN with a Recurrent Neural Network (RNN) to recognize spoken language in seven different languages. The technique used leverages the advantages of these architectures to construct the CRNN model. Ultimately, the speech signal was designated as a frame and employed as the input for the CRNN model. Alashban and Alotaibi [13] propose a deep learning system, specifically Bidirectional Long Short-Term Memory (BiLSTM), for identifying and differentiating Arabic from other similar languages taken from the Mozilla speech corpus. Furthermore, the study employs a linguistic analysis of these languages. The Bi-LSTM model is implemented and designed using acoustic signal properties.

Spoken Language Identification (SLID) is a critical task in speech processing that involves determining the language of a given speech utterance. Traditional SLID approaches rely on phonotactic, prosodic, and spectral features to differentiate languages. Early methods employed Gaussian Mixture Models (GMMs) and Hidden Markov Models (HMMs) for classification, but these techniques struggled with robustness in real-world conditions. Rawat et al. [14] describes a method for Automatic Speech Recognition (ASR) that utilizes the Whale Optimized Random Forest Algorithm (WO-RFA) to enhance the accuracy of speech recognition. The DBODL-MSLIS approach combines the advantages of the Random Forest (RF) algorithm with the optimization capabilities of the Whale Optimizer Algorithm (WOA).

Chithra Madhu et al. proposed an automatic language identification approach for seven different languages [15]. They use phonotactic as well as prosodic features. In the phonotactic approach, a multilingual phonetic engine is used to obtain the phoneme sequence of the input speech utterance. In the prosodic approach, feature vectors were obtained by concatenating the syllables. A feed forward neural network is used as a classifier for obtaining the language identity from given speech utterance. Jebali et al. [16] recommend a deep convolutional LSTM model that simultaneously exploits the non-manual features. With advancements in deep learning, Convolutional Neural Networks (CNNs), Recurrent Neural Networks (RNNs), and Transformer-based models have significantly improved SLID performance. Mukherjee et al. focused on identifying seven Indic languages using the IIIT-H Indic Speech Databases by analyzing their frequency envelope textures [17]. This task is particularly challenging due to the overlap in word syllables and frequency patterns across different languages. The study employed deep learning techniques, specifically convolutional neural networks (CNNs), to characterize these languages, demonstrating improved performance compared to traditional approaches. Lopez-Moreno et al. [18] presented a deep neural network (DNN)-based approach for spoken language identification. They used phoneme posterior probabilities across different languages, combining DNNs with i-vector representations. The network architecture included hidden layers with 2560 neurons using ReLU activation and a softmax output layer. They tested the model on datasets such as LRE’09 and the Google 5M Language Identification corpus. This work established a strong foundation for applying deep learning techniques in SLID tasks. Sarthak, Shukla, and Mittal (2019) explored the use of convolutional neural networks (ConvNets) for spoken language identification [19]. Their work highlights the ability of ConvNets to automatically extract and learn discriminative features from speech data without relying heavily on handcrafted features. By leveraging deep learning, their approach effectively handled the complexities of speech signals, showing potential for application in multilingual speech processing tasks. Ruan van der Merwe [20] proposed a method aimed at improving the generalization of SLID for short speech utterances by using a combination of triplet loss and cross-entropy loss (CEL), referred to as triplet entropy loss. This method utilized a convolutional neural network architecture derived from the pretrained ResNet50 model. The architecture included a softmax activation in the final layer. To optimize training, the model used the Adam optimizer with a learning rate of 10^−4^, a batch size of 32, and ridge-based regularization to help reduce overfitting. The highest accuracy achieved was 78% when using triplet loss. Badr M. Abdullah et al. introduced a deep neural network-based model designed to identify Slavic languages and other linguistically similar languages [21]. The model architecture consists of two main components: a segment-level feature extractor and a language classifier. It uses a convolutional neural network (CNN) with layers containing 128, 256, and 512 filters, and kernel sizes of 5, 10, and 10, respectively, with a stride of 1 in each layer. The authors evaluated the model using two approaches: Baseline LID and Robust LID. The Baseline LID approach achieved an average accuracy of 53.25%, while the Robust LID approach significantly improved performance, reaching an average accuracy of 87.32%. Xugang Lu et al. present an unsupervised neural network-based model for SLID that aims to reduce distribution variance across both features and classifiers between training and testing datasets [22]. To address this, the study employs the optimal transport (OT) method to quantify distribution differences. Additionally, a Time Delay Neural Network (TDNN) framework is used to facilitate adaptation between the training and test sets. Pardeep Rangan et al. [23] proposed a framework that integrates a Convolutional Neural Network (CNN) with a Long Short-Term Memory (LSTM) network, using the Connectionist Temporal Classification (CTC) loss function. In this approach, speech signals are first transformed into spectrograms. CNN layers are then applied to extract relevant features, followed by LSTM layers that capture temporal dependencies from the extracted features. The proposed method achieved an accuracy ranging from 74% to 76%. Mudit Verma and Arun Balaji proposed a capsule network framework for identifying spoken language identification systems [24]. In CapsNet, a convolutional layer has a total of 128 kernels with the size of (9,9,1) and a step size of 1 with ReLU function. It divided the CapsNet into two parts: encoder and decoder. The first 4 layers represent the encoder, and the last 3 layers represent the decoder. They achieved an accuracy of 91.80% with 5 s audio clips using the CapsNet approach. Surabhi Punjabi et al. [25] proposed a Recurrent Neural Network Transducer (RNN-T) for speech recognition and spoken language identification. They use two languages pairs: English–Spanish and English–Hindi. The RNN-T framework uses 5 encoder LSTM layers with 1024 units and 2 decoder LSTM layers with 1024 units; a 512-d embedding layer was used as a decoder input. The discrete fracture network (DFN) has 512 hidden neurons, along with tanh and softmax functions.

Ravi Kumar Vuddagiri et al. [26] proposed a curriculum learning-based framework for noise-robust language identification (LID) using Deep Neural Networks with Attention (DNN-WA). The study addressed performance degradation in noisy environments by training multi-SNR models with a structured progression of noise complexity. i-vector, DNN, and DNN-WA architectures were evaluated using the IIIT-H Indian database. The work highlights the advantage of curriculum learning in SLID systems that generalize well across diverse acoustic conditions. More recently, biomimetic computing approaches, inspired by natural intelligence, have emerged as powerful optimization techniques for feature selection and classification.

## 2. Material and Methods

**Figure 1:**
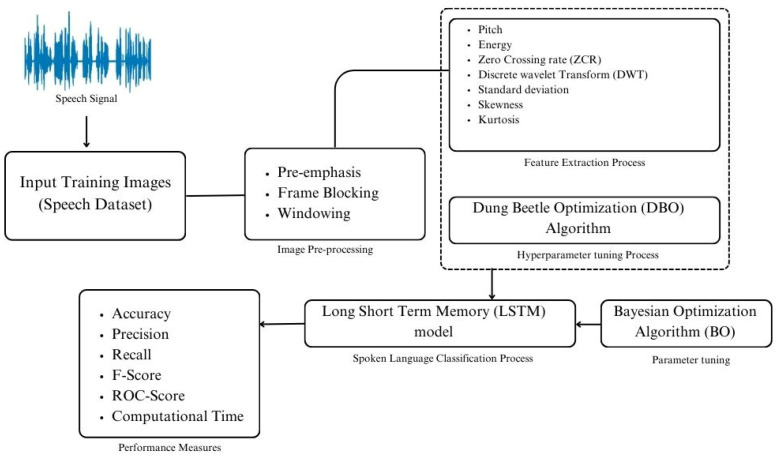
Workflow of DBODL-MSLIS technique.

Figure 1 illustrates the workflow of the proposed DBODL-MSLIS technique to detect and classify multiple spoken languages using speech signals. It consists of following four key processes involved: preprocessing, feature extraction, Dung Beetle Optimization algorithm for hyperparameter tuning, and finally the LSTM-based classification. The features were extracted using Python (version 3.12.7; Python Software Foundation, Wilmington, DE, USA) within the Anaconda platform (version 2024.10-1; Anaconda Inc., Austin, TX, USA). The processing was carried out in Jupyter Notebook (Project Jupyter, Berkeley, CA, USA), using the Librosa (version 0.10.0.post2; librosa.org) and SciPy (version 1.14.1; SciPy Community, USA) libraries for feature extraction.

### 2.1. Preprocessing

**Figure 2:**
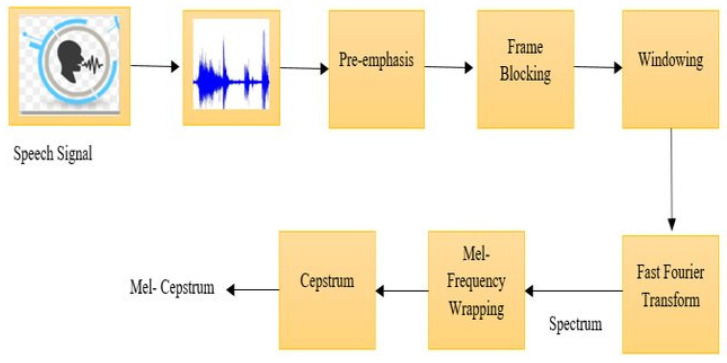
Process flow for speech signal preprocessing [28].

As a first step, the DBODL-MSLIS technique incorporates speech preprocessing, which includes a series of consecutive steps in the following order: pre-emphasis, frame blocking and windowing (Figure 2) [27]. The preprocessing phase involved cleaning and normalizing the audio data, converting speech signals into spectrograms, and extracting Mel-Frequency Cepstral Coefficients (MFCCs). This ensured the model was fed high-quality features, improving classification accuracy. The data were also standardized to have a mean of 0 and a variance of 1 to enhance training stability.

The preprocessing begins with pre-emphasis, where a high-pass filter is for the enhancement of high-frequency components. It helps to improve speech clarity. Next, signal framing breaks the continuous speech signal into fixed-length frames of 20 ms, with a 10 ms overlap to preserve temporal continuity. Finally, a Hamming window is used to reduce the spectral leakage at frame boundaries to minimize signal discontinuities.

### 2.2. Feature Extraction

In spoken language identification (SLID), selecting the appropriate set of acoustic and phonetic features is crucial for accurately capturing the distinctive characteristics of each language. These features provide the necessary foundation to differentiate spoken languages from the given speech signals. In the proposed method, we focus on a diverse combination of time-domain and frequency-domain features—such as pitch, energy, zero crossing rate (ZCR), discrete wavelet transform (DWT), standard deviation, skewness, and kurtosis [29]. Each of these features offers valuable insight into the rhythm, tone, and statistical properties of speech. Below is a brief explanation of each, highlighting their importance and function within our proposed system.

Pitch: The pitch represents the ear’s response to the sound wave frequency. An auto-correlation technique to measure pitch, which ensures precise analysis of variation in speech across different languages.

Energy: It is the measure of a sound’s strength as perceived by the human ear, which depends on the wave’s amplitude. A higher amplitude results in a louder sound. This feature is extracted using the Fast Fourier Transform (FFT), which helps analyze the signal’s frequency components for language classification.

Zero crossing rate (ZCR): It is a measure of the rate at which a signal changes from positive values to zero to negative values. It is a significant feature to detect the presence of human speech, especially for finding the difference between voiced and unvoiced sounds. ZCR identify short, high energy burst in speech signal for language identification.

To extract meaningful features for SLID, several statistical and transform-based techniques have been used, including discrete wavelet transform (DWT), standard deviation, skewness, and kurtosis.

Discrete Wavelet Transform (DWT): DWT decomposes signals into frequency subbands, where the higher frequency band captures detailed coefficients and the lower frequency band contains approximate coefficients. The Haar wavelet is used to analyze local signal features.

Standard Deviation: Standard deviation of important acoustic features such as pitch, formants, and spectral coefficients helps measure their variability within each language class.

Skewness: Skewness is analyzed to detect any biases or imbalances in the distribution of acoustic features across different languages, which helps improve the robustness of classification models

Kurtosis: Kurtosis is used to estimate the deviation from the Gaussian distribution in acoustic features. A higher value of kurtosis indicates stronger peaks in feature distribution.

The combination of extracted features significantly strengthens the classification ability of the proposed spoken language identification model. Features from the time domain, such as zero crossing rate (ZCR) and pitch, help in capturing prosodic details like rhythm and intonation. On the other hand, frequency-domain features like energy and the discrete wavelet transform (DWT) focus on identifying frequency-specific variations in speech. Additionally, statistical measures—standard deviation, skewness, and kurtosis—highlight differences in data spread, asymmetry, and peak concentration, which are often language-dependent. Together, these features offer a well-rounded representation of speech signals, enabling the proposed method to perform accurately and consistently across diverse multilingual datasets.

### 2.3. Feature Selection Using DBO Algorithm

Feature selection plays a vital role in reducing overfitting, improving model performance, and minimizing computational time. By selecting only the most relevant features, we enhance model interpretability and efficiency, making it better suited for real-world applications.

Traditional feature selection methods [30] fall into three categories:

- Filter methods (e.g., mutual information, correlation) are fast but ignore feature dependencies.
- Wrapper methods (e.g., recursive feature elimination) offer better accuracy but are computationally expensive.
- Embedded methods (e.g., LASSO, decision trees) combine selection and learning but can sometimes overfit.

In this work, we employ the DBO algorithm to strike a balance between performance and efficiency. It is well-suited for high-dimensional data, like that in SLID, and efficiently explores the feature space without requiring exhaustive searches. The DBO algorithm is inspired by the natural behaviors of dung beetles, such as rolling, dancing, foraging, reproduction, and stealing. These behaviors are mathematically modeled to guide the feature selection process and help to identify optimal solutions.

The rolling behavior in DBO simulates dung beetles navigation using environmental cues such as sunlight or wind. For SLID tasks, it guides exploration by adjusting feature subsets based on past positions and distance from the worst solution, as shown in Equation (1).

(1) $$x_{r}(t+1)=x_{r}(t)+\beta \times e\times x_{r}\left(t-1\right)+b\times \Delta x\;\Delta x=\left|x_{r}\left(t\right)-X^{worst}\right|$$

For this particular instance, the position of the database at iteration t is denoted by $x_{r}\left(t\right)$, the orientation coefficient is denoted by β, the deflection coefficient is denoted by e, and the global worst position is denoted by $X^{worst}$.

DBs exhibit dancing behavior when faced with barriers, wherein they engage in a process of recovery and pathfinding. In the SLID feature selection process, this behavior helps the algorithm to recover from less effective feature subsets and redirect the search toward more optimal solutions. This behavior is mathematically represented by the equation

(2) $$x_{r}\left(t+1\right)=x_{r}\left(t\right)+tan\;\alpha \left|x_{r}\left(t\right)-x_{r}\left(t-1\right)\right|$$

where α represents the angle of direction selected.

The reproductive behavior in the Dung Beetle Optimization algorithm mirrors the way female dung beetles choose safe spots near optimal locations to lay their eggs. In our application, reproductivity is modeled by generating new feature subsets around the current best solution. This strategy helps to intensify the search in high-potential regions of the feature space, increasing the chances of selecting the most relevant features. The behavior is mathematically represented by

(3) $$Ls=max\left\{X^{best}\times \left(1-A\right),Lb\right\},\;Us=min\left\{X^{best}\times \left(1+A\right),Ub\right\}$$

(4) $$x_{B}\left(t+1\right)=X^{best}+B_{1}\times \left(x_{B}\left(t\right)-Ls\right)+B_{2}\times \left(x_{B}\left(t\right)-Us\right)$$

The foraging behavior in the DBO algorithm is inspired by young dung beetles emerging from underground to search for food within safe zones. This behavior supports the exploration of new feature subsets within defined boundaries around the global best solution.

(5) $$Lf=\max\left\{X^{Gbest}\times \left(1-A\right),Lb\right\},\;Uf=min\left\{X^{Gbest}\times \left(1+A\right),Ub\right\}$$

(6) $$x_{\gamma }\left(t+1\right)=x_{\gamma }\left(t\right)+Q_{1}\times \left(x_{\gamma }\left(t\right)-Lf\right)+Q_{2}\times \left(x_{\gamma }\left(t\right)-Uf\right)$$

The stealing behavior in DBO mimics dung beetles robbing resources near optimal spots. For language identification process, it explores feature subsets around the global best solution $X^{Gbest}$, improving convergence toward highly relevant features.

(7) $$x_{s}\left(t+1\right)=X^{Gbest}+S\times E\times \left(\left|x_{s}\left(t\right)-x^{worst}\right|+\left|x_{s}\left(t\right)-X^{Gbest}\right|\right)$$

Finally, the fitness function (FF) utilized in the DBO system strikes a balance between the number of selected features and classifier accuracy:

(8) $$Fitness=\alpha \gamma_{R}\left(D\right)+\beta \frac{\left|R\right|}{\left|C\right|}$$

The classifier error is represented by $\gamma_{R}\left(D\right)$, the count of selected features is denoted by ∣R∣, ∣C∣ is the total number of features, and α and β are parameters that balance classifier quality and subset length.

DBO helps in selecting the most relevant features to improve the classification performance of SLID. Equations (1)–(8) explain how the DBO algorithm works using the key behaviors like rolling, dancing, foraging, reproducing, and stealing. In rolling behavior, feature vectors adjust their positions by moving away from the worst solutions $X^{worst}$ toward more promising regions in the feature space. Dancing behavior introduces directional shifts (α) to escape unproductive regions and explore new areas that may provide better feature combinations. Reproductive behavior generates new feature subsets between the lower (Ls) and upper (Us) bounds, targeting on top-performing features. Stealing behavior allows weaker subsets to adopt useful features from stronger ones ${(X}^{Gbest}).$ The fitness function balances classification accuracy $\gamma_{R}\left(D\right)$ and feature reduction ($\frac{\left|R\right|}{\left|C\right|}$), resulting in a compact and high-performing feature set for SLID.

### 2.4. Spoken Language Classification Using LSTM with Bayesian Optimization

An LSTM model combined with Bayesian Optimization (BO) is used to classify spoken languages by capturing patterns and dependencies in sequential data.

#### 2.4.1. LSTM Model

Long Short-Term Memory (LSTM) networks are highly suitable for Spoken Language Identification (SLID) tasks because of their capability to understand and retain patterns over time in speech data. Unlike standard neural networks, LSTMs include specialized memory units that help preserve context across long sequences, which is essential for processing the continuously changing nature of spoken input. For SLID, LSTMs use a set of gates—namely input, forget, and output gates—to manage how information is stored, removed, or passed through the network. This gate-based structure allows the model to focus on meaningful features and filter out less useful data [31]. Such a mechanism is especially beneficial when trying to distinguish between languages that may share similar sounds or intonations. By maintaining and updating memory states as new input arrives, LSTMs effectively learn long-term dependencies, leading to improved recognition and classification performance. Their strength in handling time-based speech variations makes them suitable for developing language identification systems.

#### 2.4.2. Bayesian Optimization

In this study, Bayesian Optimization (BO) was employed to efficiently optimize key hyperparameters of the LSTM model—such as learning rate, batch size, and the number of hidden units—in order to reduce classification errors in SLID. Compared to conventional methods like grid search or random search, BO offers a more efficient solution by exploring fewer configurations while still locating optimal parameter combinations [32]. It constructs a probabilistic model of the objective function, commonly using a Gaussian process, and applies the Expected Improvement (EI) acquisition function to guide the search for the most promising hyperparameters. This method ensures a balanced approach between discovering new regions of the search space and exploiting areas known to yield good performance. In the context of SLID, BO enhanced both accuracy and generalization by integrating regularization techniques such as dropout and early stopping to prevent overfitting. Furthermore, 5-fold cross-validation was used to validate model stability across different data splits. Overall, BO demonstrated its effectiveness in optimizing deep learning models for large-scale speech data, leading to improved performance with reduced computational overhead.

## 3. Results

The performance validation of the proposed method is conducted using the benchmark IIIT Spoken Language dataset [33] which comprises speech and textual data for the following Indian languages: Hindi, Bangla, Kannada, Malayalam, Marathi, Tamil, and Telugu. Each of the languages selected has over 10,000 articles on Wikipedia, ensuring a rich linguistic dataset [34]. It includes 7000 audio instances, evenly distributed across the seven language classes mentioned, with 1000 samples per language [35,36]. The duration of each speech segment ranges from 1 to 3 s. To filter out silent segments and enhance the quality of data, voice activity detectors were applied. Native speakers recordings are used in the data to ensure the dialectical authenticity and reliability [37].

In this work, we focus on identifying spoken languages using a combination of extracted audio features and machine learning models. The features like Pitch, Energy, Zero Crossing Rate (ZCR), and Discrete Wavelet Transform (DWT) was extracted using standard Python (version 3.12.7) within the Anaconda environment, utilizing libraries such as Librosa and SciPy (version 1.14.1) through Jupyter Notebook.LSTM networks was implemented with both Keras and Tensorflow for classification. To incorporate uncertainty in predictions and improve reliability, we also explore Bayesian Neural Networks with the help of TensorFlow Probability. Hyperparameters are fine-tuned using the Dung Beetle Optimization (DBO) algorithm, implemented using the mealpy library. Finally, the models are evaluated using accuracy, F1-score, and confusion matrices, with visualization and analysis carried out using tools from scikit-learn and seaborn.

**Figure 3:**
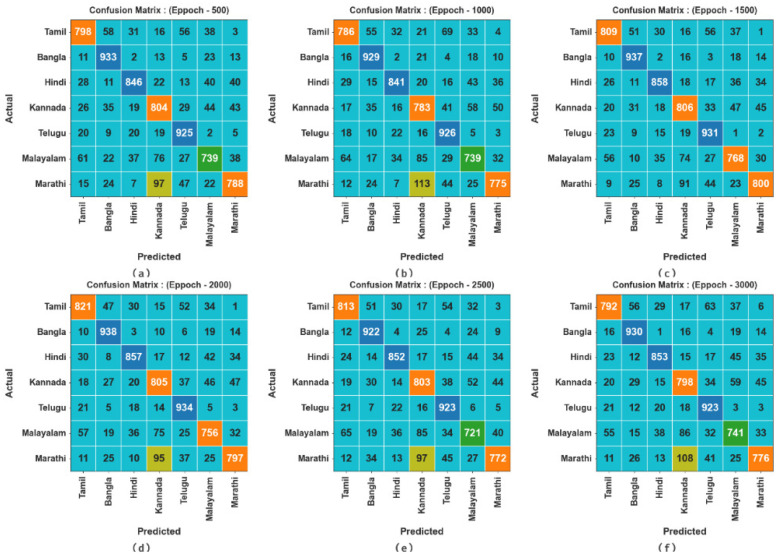
Confusion matrices of DBODL-MSLIS technique (**a**–**f**) Epochs 500–3000.

Figure 3 illustrates the confusion matrices attained by the DBODL-MSLIS technique for seven spoken languages at different training stages. A confusion matrix provides insight into the performance of the DBODL-MSLIS model by visualizing classification errors.

**Figure 4:**
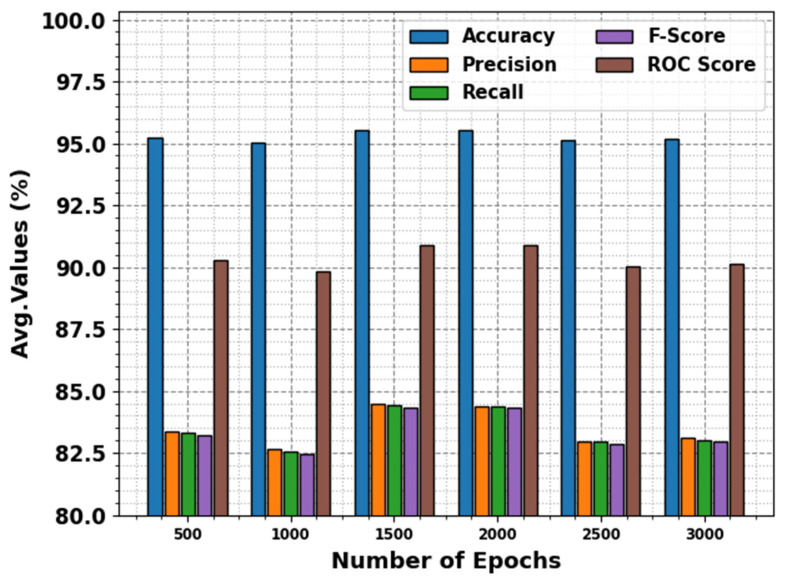
Average of DBODL-MSLIS algorithm under several epochs.

We have obtained the evaluation matrices such as accuracy, precision, recall, F-score, and the ROC curve for different epochs as shown in Table 1 and Figure 4. The results portrayed that the DBODL-MSLIS technique properly identified all the spoken languages. The best results are obtained with 1500 epochs, with average $accu_{y}$ of 95.55%, $prec_{n}$ of 84.46%, $reca_{l}$ of 84.41%, $F_{score}$ of 84.34%, and $ROC_{score}$ value of 90.91%.

**Figure 5:**
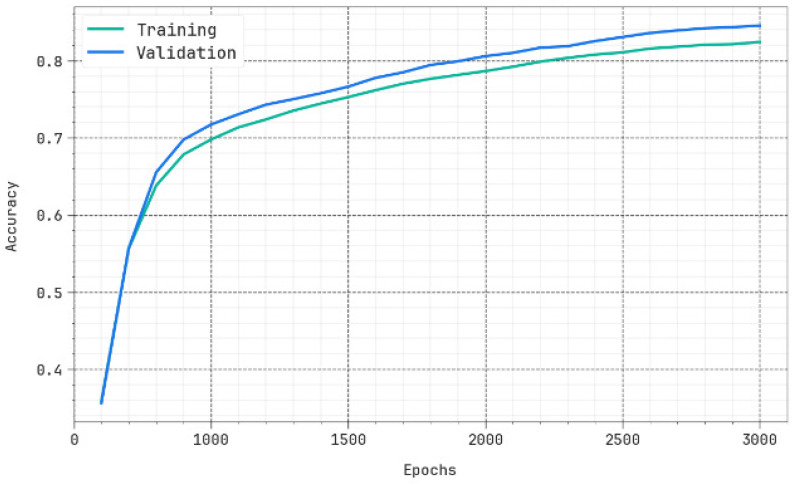
$Accu_{y}$ curve of DBODL-MSLIS technique.

**Figure 6:**
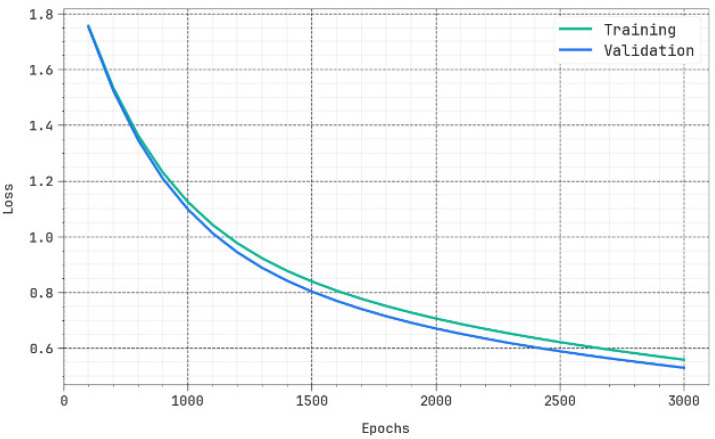
Loss curve of DBODL-MSLIS technique.

The efficiency of the proposed method in terms of accuracy and loss for the training and validation data is shown in Figure 5 and Figure 6, respectively. It is clearly evident from the figure that the accuracy/loss does not decrease much for the validation data and hence depicts the generalization capability of the method.

**Figure 7:**
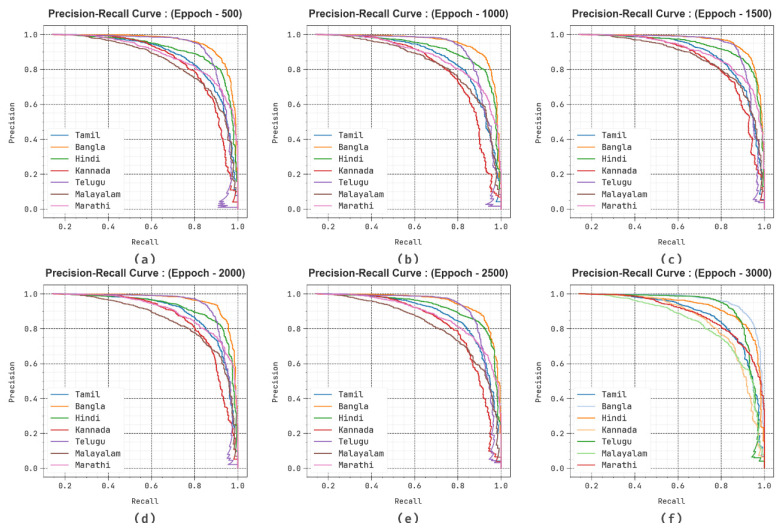
PR curve of DBODL-MSLIS technique (**a**–**f**) Epochs 500–3000.

The generalization capability of the method can be examined with the help of Precision–Recall (PR) curve as revealed in Figure 7. The experimental outcome shows that the DBODL-MSLIS approach progressively completes superior rates of PR with seven classes at several epochs.

**Figure 8:**
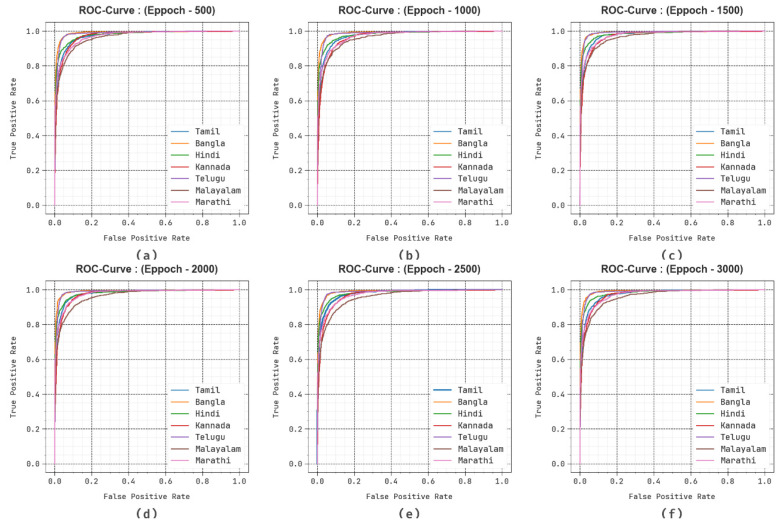
ROC curve of DBODL-MSLIS system (**a**–**f**) Epochs 500–3000.

Furthermore, in Figure 8, the ROC curves obtained by the DBODL-MSLIS algorithm outperformed the classifiers of various labels at multiple epochs. It provides a comprehensive understanding of the trade-off between True positive rate (TPR) and False positive rate (FPR) at various exposure threshold rates and epochs.

**Figure 9:**
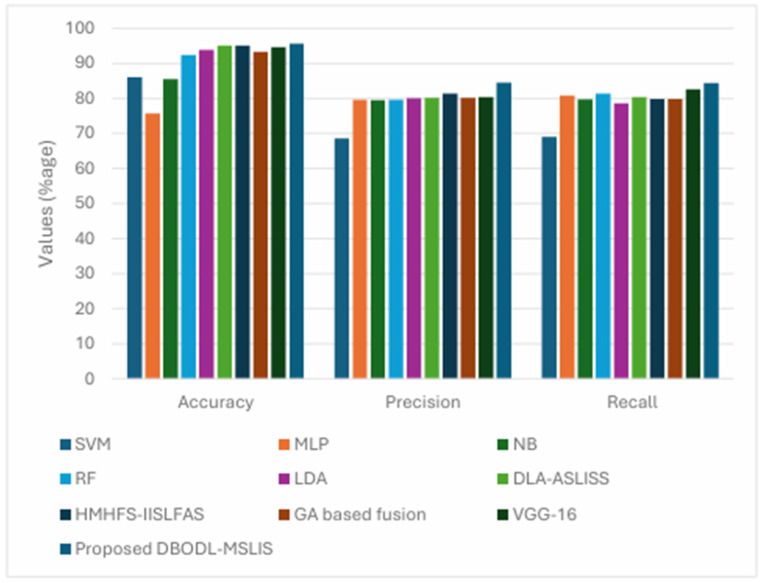
Comparative outcome of DBODL-MSLIS technique with state of the art approaches.

The comparative analysis of the proposed model with the other state of the art model commonly used for spoken language identification is shown in Table 2 and Figure 9. Several state-of-the-art models were used for performance comparison in the SLID task. The Support Vector Machine (SVM) model employed a radial basis function (RBF) kernel with a regularization parameter C = 1 and gamma set to ‘scale’.

The Multi-Layer Perceptron (MLP) consisted of an input layer followed by two hidden layers: L_1_ with 64 neurons (ReLU activation) and L_2_ with 32 neurons (ReLU), leading to a 7-class softmax output layer. The Gaussian Naïve Bayes (NB) classifier was used as a simple probabilistic baseline. The Random Forest (RF) model consisted of 100 trees, used Gini impurity for splitting, and was initialized with a random state of 42. The Linear Discriminant Analysis (LDA) model was implemented using singular value decomposition.

In addition, several hybrid and deep learning-based models were evaluated. The DLA-ASLISS model combined CNN and LSTM architectures with a structure similar to the MLP above, incorporating dropout (rate 0.5) to prevent overfitting. The HMHFS-IISLFAS model used an MLP optimized via Particle Swarm Optimization (PSO) for feature selection and model tuning. The GA-based Fusion model combined acoustic (ALID) and phonetic (PLID) information using a Genetic Algorithm for effective feature fusion. The VGG-16 + BiLSTM architecture integrated a 16-layer deep CNN (13 convolutional and 3 fully connected layers), using 3 × 3 convolution kernels and ReLU activations, followed by a BiLSTM for temporal modeling.

Finally, the proposed DBODL-MSLIS model combined LSTM and BO with Dung Beetle Optimization for feature selection. Its architecture included an input layer, followed by two hidden layers (64 and 32 neurons with ReLU activations), and a final softmax output layer. The model was trained for 3000 epochs with a learning rate of 0.001, demonstrating strong performance in optimizing hyperparameters and minimizing classification errors. The proposed technique accomplishes superior results with enhanced $accu_{y}$ of 95.55%, $prec_{n}$ of 84.46%, and $reca_{l}$ of 84.41%.

In addition to the strong performance achieved using the DBODL-MSLIS framework, it is also valuable to reflect on how this approach compares with other state-of-the-art biomimetic computing models like Particle Swarm Optimization (PSO) and Ant Colony Optimization (ACO).

**Figure 10:**
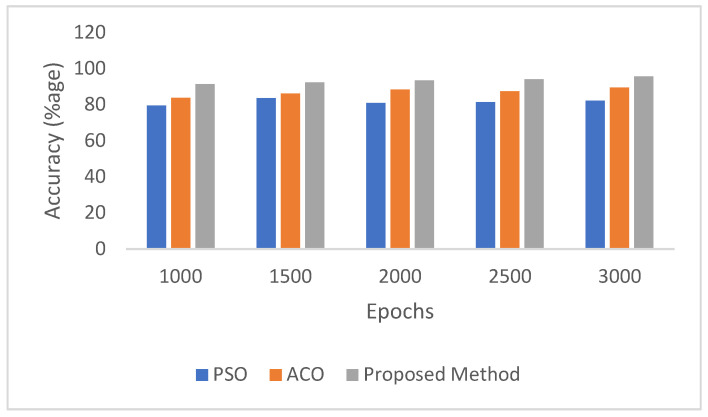
Comparative accuracy of state-of-the-art biomimetic computing models.

Table 3 and Figure 10 illustrate the classification accuracy obtained by these methods over varying training epochs. PSO demonstrates moderate performance, with accuracy peaking at 83.45% after 1500 epochs and fluctuating thereafter. ACO exhibits more consistent improvement, reaching a maximum accuracy of 89.45% at 3000 epochs. In contrast, the proposed DBODL-MSLIS method, which integrates Bayesian Optimization with LSTM, consistently outperforms both PSO and ACO across all epochs, culminating in a highest accuracy of 95.55%. These results underline the robustness and effectiveness of the proposed model in enhancing spoken language identification through superior hyperparameter optimization.

## 4. Discussion and Conclusions

This study presented the DBODL-MSLIS method, a biomimetic-inspired system designed for effective and accurate identification of spoken languages from speech signals. The approach integrates several key stages: speech preprocessing, comprehensive feature extraction, feature selection using the Dung Beetle Optimization (DBO) algorithm, and classification via a Long Short-Term Memory (LSTM) model enhanced through Bayesian Optimization (BO). The extracted features—such as pitch, energy, Discrete Wavelet Transform (DWT), and Zero-Crossing Rate (ZCR)—were used to capture essential patterns in speech, while the DBO algorithm efficiently filtered out redundant information to strengthen classification accuracy.

To assess its performance, the IIIT-Hyderabad Spoken Language Identification dataset, which includes a variety of Indian languages, was employed. The DBODL-MSLIS framework achieved strong results, with an average accuracy of 95.55%, precision of 84.46%, recall of 84.41%, F-score of 84.34%, and a ROC-AUC score of 90.91%. These findings confirm that the proposed method performs significantly better than many existing models. The results also demonstrate that DBODL-MSLIS generalizes effectively across different speech inputs, ensuring both high reliability and accuracy in multilingual language identification tasks.

## Figures and Tables

**Table 1:** Outcome of DBODL-MSLIS algorithm at several epochs.

Classes	$Accu_{y}$	$Prec_{n}$	$Reca_{l}$	$F_{Score}$	$ROC_{Score}$
Epoch—500					
Tamil	94.81	83.21	79.80	81.47	88.56
Bangla	96.77	85.44	93.30	89.20	95.33
Hindi	96.14	87.94	84.60	86.24	91.33
Kannada	93.73	76.79	80.40	78.55	88.18
Telugu	96.40	83.94	92.50	88.01	94.78
Malayalam	93.86	81.39	73.90	77.46	85.54
Marathi	94.94	84.73	78.80	81.66	88.22
Average	95.24	83.35	83.33	83.23	90.28
Epoch—1000					
Tamil	94.71	83.44	78.60	80.95	88.00
Bangla	96.76	85.62	92.90	89.11	95.15
Hindi	96.11	88.16	84.10	86.08	91.11
Kannada	92.96	73.94	78.30	76.06	86.85
Telugu	96.04	82.02	92.60	86.99	94.61
Malayalam	93.67	80.24	73.90	76.94	85.43
Marathi	94.86	85.16	77.50	81.15	87.62
Average	95.02	82.65	82.56	82.47	89.82
Epoch—1500					
Tamil	95.21	84.89	80.90	82.85	89.25
Bangla	97.14	87.24	93.70	90.36	95.71
Hindi	96.43	88.82	85.80	87.28	92.00
Kannada	93.89	77.50	80.60	79.02	88.35
Telugu	96.44	83.80	93.10	88.20	95.05
Malayalam	94.37	82.58	76.80	79.59	87.05
Marathi	95.34	86.39	80.00	83.07	88.95
Average	95.55	84.46	84.41	84.34	90.91
Epoch—2000					
Tamil	95.34	84.81	82.10	83.43	89.82
Bangla	97.24	87.75	93.80	90.67	95.81
Hindi	96.29	87.99	85.70	86.83	91.87
Kannada	93.99	78.08	80.50	79.27	88.37
Telugu	96.64	84.68	93.40	88.83	95.29
Malayalam	94.07	81.55	75.60	78.46	86.38
Marathi	95.23	85.88	79.70	82.68	88.76
Average	95.54	84.39	84.40	84.31	90.90
Epoch—2500					
Tamil	95.14	84.16	81.30	82.71	89.38
Bangla	96.67	85.61	92.20	88.78	94.81
Hindi	96.19	87.74	85.20	86.45	91.61
Kannada	93.51	75.75	80.30	77.96	88.01
Telugu	96.19	82.93	92.30	87.36	94.57
Malayalam	93.37	79.58	72.10	75.66	84.51
Marathi	94.81	85.12	77.20	80.96	87.48
Average	95.13	82.98	82.94	82.84	90.05
Epoch—3000					
Tamil	94.94	84.43	79.20	81.73	88.38
Bangla	96.86	86.11	93.00	89.42	95.25
Hindi	96.24	88.03	85.30	86.64	91.68
Kannada	93.40	75.43	79.80	77.55	87.73
Telugu	96.17	82.85	92.30	87.32	94.56
Malayalam	93.61	79.76	74.10	76.83	85.48
Marathi	94.86	85.09	77.60	81.17	87.67
Average	95.16	83.10	83.04	82.95	90.11

**Table 2:** Comparative outcome of DBODL-MSLIS technique with recent approaches.

Model	SVM	MLP	NB	RF	LDA	DLA-ASLISS	HMHFS-IISLFAS	GA Based Fusion	VGG-16	Proposed DBODL-MSLIS
Accuracy	86	75.7	85.48	92.35	93.88	95	95.11	93.3	94.6	95.55
Precision	68.47	79.59	79.48	79.62	79.97	80.13	81.31	80.11	80.33	84.46
Recall	69	80.78	79.7	81.32	78.57	80.34	79.75	79.82	82.55	84.41

**Table 3:** Comparative accuracy analysis for proposed model.

Epochs	1000	1500	2000	2500	3000
PSO	79.44	83.45	80.79	81.33	82.13
ACO	83.67	86.13	88.22	87.33	89.45
Proposed DBODL-MSLIS	91.25	92.15	93.27	94.03	95.55

## Data Availability

The datasets generated and/or analyzed during the current study are available from the corresponding author on reasonable request. Publicly available datasets were used in this study. These data can be found at https://www.kaggle.com/datasets/sizlingdhairya1/iiit-spoken-language-datasets (accessed on 21 October 2024).
